# Anticancer efficacy of hirsuteine against colorectal cancer by opposite modulation of wild-type and mutant p53

**DOI:** 10.1007/s12672-023-00688-1

**Published:** 2023-05-31

**Authors:** Yan Zhang, Tingting Guo, Shurong Li, Zehao Ren, Shan Gao, Hao Lu, Xuelan Ma, Donghui Liu, Yao Liu, Dexin Kong, Yuling Qiu

**Affiliations:** 1 Tianjin Key Laboratory on Technologies Enabling Development of Clinical Therapeutics and Diagnostics, School of Pharmacy, Tianjin Medical University, Tianjin, China; 2 Department of Otorhinolaryngology Head and Neck Surgery, Tianjin First Central Hospital, Tianjin, China

**Keywords:** Hirsuteine, Opposite modulation, Wild-type p53, Mutant p53, Colorectal cancer

## Abstract

**Purpose:**

The present study aimed to explore the anticancer activity of hirsuteine (HST), an indole alkaloid from the traditional Chinese herbal medicine *Uncaria rhynchophylla*, against colorectal cancer (CRC) and the underlining mechanism.

**Methods:**

MTT, colony formation, flow cytometry and MDC staining were conducted to confirm the antiproliferative effect of HST on human CRC cells harboring different p53 status. Protein expressions were evaluated by the Western blot analysis. p53 protein half-life and the interaction between p53 and MDM2 were investigated using cycloheximide (CHX)-chase assay and Co-immunoprecipitation (Co-IP), respectively. Transcriptional activity of p53 was examined by qRT-PCR and Chromatin immunoprecipitation (ChIP). Xenograft tumor in nude mice was created to evaluate in vivo anticancer effect of HST against CRC.

**Results:**

HST inhibited cell growth, arrested cell cycle and induced autophagy, showing efficient anticancer effects on CRC cells independent of p53 status. In HCT-8 cells, HST prolonged wtp53 half-life, and upregulated mRNA level of p21, suggesting that HST activated the p53 pathway through enhancement of wtp53 stability and transcriptional activity. Meanwhile in SW620 cells, HST induced MDM2-mediated proteasomal degradation of mutp53^R273H^, increased the DNA-binding ability of mutp53^R273H^ at the p21 promoter, and upregulated mRNA levels of p21 and MDM2, demonstrating the depletion of mutp53^R273H^ and restoration of its wild-type-like properties by HST. p53 knockdown by siRNA significantly impaired the growth inhibition of HST on HCT-8 and SW620 cells. Moreover, HST showed anticancer effects in xenograft tumors, accompanied with an opposite regulation of wtp53 and mutp53 ^R273H^ in mechanism.

**Conclusion:**

This study revealed the anticancer efficacy of HST against CRC via opposite modulation of wtp53 and mutp53 ^R273H^, indicating the potential of HST to be a CRC drug candidate targeting p53 signaling.

**Supplementary Information:**

The online version contains supplementary material available at 10.1007/s12672-023-00688-1.

## Introduction

Colorectal cancer (CRC) is currently one of the leading causes of cancer deaths throughout the world and ranks the fourth deadliest cancer type among various cancers [1, 2]. Due to changes in lifestyle and less physical exercise, the incidence of CRC has increased continuously, especially in developing countries [2]. Chemotherapy based on 5-Fu or 5-Fu combined with other adjuvants or targeted agents are still the main drug treatment strategies. Despite advances in targeted chemotherapies, such as cetuximab and bevacizumab, having greatly improved the CRC clinical response over the past few decades, disease relapse and therapeutic resistance are still problems that urgently need to be resolved [3, 4].

Through transcriptional regulation of multiple genes, wild-type p53 (wtp53), which is coded by *TP53*, plays significant roles in tumorigenesis as an important tumor-suppressor [5]. However, it has been investigated that *TP53* is frequently mutated in more than 50% of human cancers, leading to mutant p53 protein (mutp53) [6]. p53 mutations not only impair the tumor suppressive functions of its wild-type but also acquire oncogenic functions, so-called gain-of-function (GOF), and contribute to the malignant characteristics, such as aggressiveness, invasiveness and chemoresistance [6]. The mutation of p53 is commonly found in ~ 60% of CRC patients. Notably, mutp53 have been frequently observed in both the distal and proximal colorectal tumors [7]. Patients carrying mutant p53 proteins display higher oncogenic potential, poor prognosis, poor response to chemotherapy and accelerated tumor recurrence [8]. In this scenario, targeting p53, either wtp53 or mutp53, has emerged as one of the attractive therapeutic opportunities in CRC therapy.

A vast majority of cancer-associated mutations in *TP53* are missense point mutations which are commonly clustered in DNA binding domain. They are categorized into two main groups: “DNA contact mutants” and “conformational mutants”. Hot-spot mutants, such as R248W and R273H, that directly cause amino acid substitutions in the DNA-binding domain and weaken the affinity of p53 and cognate DNA, are referred to as DNA contact mutants. Other hot-spot mutants, such as R175H, G245S, and R282W, that alter the residues within the core domain and destabilize the protein conformation, belong to the category of conformational mutants [5, 9]. Studies have suggested that mice with the homozygous mutant of p53 R273H and R248W manifest an increased risk for metastatic cancers [10]. Researches have also demonstrated that mutp53^R273H^ plays pivotal roles in the enhanced proliferation, acquisition of stemness, drug resistance, and promotes CRC progression [8, 11].

Recently, with the tremendous clinical progression of a variety of drugs derived from medicinal plants, traditional Chinese medicines have become promising sources for novel therapeutics in the management of various diseases, including cancer. To date, indole alkaloids and their derivatives are commonly applied clinically in cancer therapy, suggesting the potential of the indole framework in anticancer drug development [12]. Hirsuteine (HST) is an indole alkaloid extracted from the traditional Chinese herbal medicine *Uncaria rhynchophylla*. As one of the main pharmacological ingredients of *Uncaria rhynchophylla,* HST has been revealed to exert anti-inflammatory, anti-oxidant, and neuromodulatory activities [13]. Several studies in the past have also demonstrated the cytotoxicity of HST on human hepatocellular carcinoma cells and breast cancer cells. Our previous study has demonstrated that HST has potential antileukemic effect through targeting sphingosine kinase 1 [14]. However, despite the valuable properties of HST, little is known regarding the anticancer activity of HST on CRC and the possible mechanism.

In the present research, we focus on the wtp53 and mutp53^R273H^ phenotype of CRC and explore the anticancer activity and mechanism of HST against CRC in vivo and in vitro.

## Materials and methods

### Reagents, antibodies and plasmids

Hirsuteine (purity: 98%) was obtained from Abphyto biotech (Chengdu, Sichuan, China). 3-(4,5-Dimethyl-2-thiazolyl)-2, 5-diphenyl-2Htetrazolium bromide (MTT) reagent was from Amresco (Solon, OH, USA). Propidium iodide (PI) was from Sigma-Aldrich (St. Louis, MO, USA). Annexin V-FITC/PI Apoptosis Detection Kit was from BD Biosciences. TRIzol reagent was purchased from Life Technologies (Carlsbad, CA, USA). Fetal bovine serum (FBS) was from Biological Industries (Kibbutz Beit-Haemek, Israel). The primary antibodies against cyclin B1 (1:1000, #4135), cdc2 (1:1000, #9116), p-Rb (1:1000, #8516), Caspase-3 (1:1000, #9662), β-actin (1:1000, #8457), anti-rabbit HRP-conjugated secondary antibodies (1:2000, #7074) and anti-mouse HRP-conjugated secondary antibodies (1:2000, #7076) were purchased from Cell Signaling Technology (Danvers, MA, USA). Antibodies against p53 (60283-1-lg), p21 (10355-1-AP), MDM2 (27883-1-AP), LC3 (14600-1-AP), p-p53 (Ser15) (67826-1-lg), p62 (18420-1-AP), Ki-67 (27309-1-AP), PUMA (55120-1-AP) were purchased from Proteintech (Wuhan, Hubei, China).

### Cell lines

Human CRC cell lines SW620 (mutp53^R273H^) and HCT-8 (wtp53) were purchased from National Collection of Authenticated Cell Cultures (Shanghai, China). Cells were maintained in complete RPMI-1640 medium supplemented with 10% FBS, 10 µg/mL streptomycin and 100 U/mL penicillin. Cells were cultured in a standard condition: a humidified incubator with 5% CO_2_ at 37 °C. Cells were authenticated by short tandem repeat (STR) analysis.

### siRNA

p53 knockdown was conducted using siRNA as our previous literature [15]. siRNA p53 was purchased from Genepharma (Suzhou, Zhejiang, China). Cells were seeded onto six-well plates (3 × 10^5^ cells/well) and transfected with siRNA p53 or siRNA non targeting using Lipofectamine^®^ 2000 (Invitrogen, Corp., Carlsbad, CA, USA) at 37 °C for 6 h. The culture medium was changed by fresh complete culture medium before subsequent experiments.

### MTT assay

The cell viability was determined by MTT assay as we previously reported [14]. Cells were seeded (4 × 10^4^ cells/well) onto 96-well plates with a volume of 200 µL per well, and treated with a series of concentrations of HST for 48 h or treated with 32 µM HST at different time points, or 32 µM HST and/or 3-MA for 48 h. After exposed with 5 mg/mL MTT, cells were further cultured at 37 °C for 4 h. The absorbance at a wavelength of 490 nm was measured by a microplate reader iMark (BIO-RAD, Hercules, CA, USA). Drug concentrations causing 50% inhibition (IC_50_ values) were calculated using GraphPad Software by interpolation.

### Colony formation

The colony formation assay was performed as previously described [16]. Cells (1 × 10^3^ cells/well) were seeded onto 6-well plates. After being treated with HST or DMSO for 48 h, the cells were further cultured for about 10 days in drug-free complete medium. Meantime, the fresh medium was changed every 3 days. The colonies were fixed with formalin and stained with 0.25% crystal violet (Sigma-Aldrich, St. Louis, MO, USA). The colonies were photographed and counted using Image J Software.

### RNA-sequencing (RNA-seq) analysis

RNA-seq analysis was conducted according to our previous literature [17]. HCT-8 cells were exposed with DMSO or HST (32 μM) for 48 h. Total RNA from cells was extracted according to qRT-PCR and prepared total RNA was then sent to Novogene Co., Ltd (Beijing, China) for sequencing and analysis. The NovoMagic tools (https://magic.novogene.com) were used for bioinformatic analysis.

### Flow cytometry

Flow cytometry was used to measure the cell cycle distribution and cell apoptosis as we reported before [17]. In brief, for cell cycle distribution analysis, after treated with HST or DMSO for 48 h, cells were collected, fixed, and permeabilized with 75% ice-cold ethanol at 4 °C overnight. Then the cells were collected and stained with propidium iodide (PI) (50 μg/mL PI, 100 μg/mL RNase A and 0.5% Triton X-100) at 4 °C for 30 min in darkness. For cell apoptosis, the treated cells were collected and stained with Annexin V/PI solution. The cell cycle distribution and cell apoptosis were further analyzed using BD Accuri C6 flow cytometer (BD Biosciences, San Jose, CA, USA).

### RNA isolation and quantitative RT-PCR

qRT-PCR was performed as our previous publication [14, 17]. Total RNA was isolated from the cells treated with HST or DMSO for 48 h using TRIzol (Life Technologies, Carlsbad, CA, USA). cDNA was synthesized from 1 μg of total RNA using StarScript II First-strand cDNA Synthesis Mix (Genstar, Beijing, China). According to the manufacturer’s instructions, qPCR was further conducted with aliquots of cDNA samples mixed with 2 × RealStar Green Fast Mixture (Genstar, Beijing, China) using a CFX96™ Real-Time PCR Detection System (BIO-RAD, Hercules, CA, USA). GAPDH was used as the reference gene for normalization using the 2^−△△Ct^ method. The sequences of primers were as follows:

- p53, Fw 5′-TAACAGTTCCTGCATGGGCGGC-3′,
- Re 5′-AGGACAGGCACAAACACGCACA-3′;
- p21, Fw 5′-TGTCCGTCAGAACCCATGC-3′,
- Re 5′-AAAGTCGAAGTTCCATCGCTC-3′;
- MDM2, Fw 5′-AGTAGCAGTGAATCTACAGGGA-3′,
- Re 5′-CTGATCCAACCAATCACCTGAAT-3′;
- GAPDH, Fw 5′-CATGAGAAGTATGACAACAGCCT-3′,
- Re 5′-AGTCCTTCCACGATACCAAAGT-3′;

### Western blotting

Western blotting was conducted as our previous publication [18]. Briefly, cells were lysed in RIPA buffer containing 1% phosphorylation inhibitors and 1% protease. Total protein was extracted, quantified, and then separated by SDS-PAGE. After electro-transfer, the polyvinylidene fluoride (PVDF) membranes were blocked, incubated with appropriate primary antibodies and HRP-conjugated secondary antibodies, and then visualized using an ECL detection kit (Thermo Fisher Scientifific, Inc., Carlsbad, CA, USA) by a ChemiDoc XRS + System (Bio-Rad, Hercules, CA, USA). β-actin was used as the internal reference.

### Monodansylcadaverine (MDC) staining

MDC staining was conducted as our previous publication to detect autophagic vacuoles induced by HST in CRC cells [18]. HCT-8 and SW620 cells were seeded in 6-well plates, exposed with HST or DMSO for 48 h, then collected and stained with MDC staining buffer (50 μM) at 37 °C for 1 h in darkness. The stained cells were washed and resuspended in Wash Buffer. The cell suspension was added to the slides and then observed by fluorescence microscopy (BX51, Olympus, Japan).

### Co-immunoprecipitation (Co-IP) assay

Co-IP assay was performed as previously described [17]. Prepared cells were lysed in lysis buffer (Beyotime, Shanghai, China) supplemented with phenylmethanesulfonyl fluoride (PMSF) and protease inhibitor cocktail at 4 °C for 30 min and pelleted by centrifugation (12,000 rpm, 20 min, 4 °C). The lysates were immunoprecipitated with p53 antibody-pretreated-beads (Protein A/G Magnetic Beads) (MCE, Princeton, NJ, USA) at 4 °C over night. Proteins were eluted from the beads and subjected to Western blot analysis as described above.

### Chromatin immunoprecipitation (ChIP) assay

The SW620 cells (3 × 10^5^ cells/mL) were seeded in 10 cm dishes, and treated with HST (32 µM) or DMSO for 48 h. ChIP assay was conducted by the ChIP Assay Kit (Beyotime, Shanghai, China) according to the manufacturer’s protocols. The cells were fixed with 1% formaldehyde at room temperature for 10 min followed by glycine solution treatment at room temperature for 5 min to stop the cross-link. The cells were collected and lysed in lysis buffer containing 1% PMSF. Cell lysates were sonicated using Bioruptor UCD-200 (Diagenode, Liège, Belgium) to obtain 200–1000 bp chromatin fragments. The sheared chromatin samples were precleared with protein A/G beads for 30 min before they were incubated with protein A/G beads and anti-p53 antibody (10442-1-AP, Proteintech, Hubei, China) at 4 °C overnight. Anti-rabbit immunoglobulin G (IgG) was used as a negative control. After extensive washing, the bead-bound immunocomplexes were eluted using elution buffer. To reverse the crosslinks, samples were treated with 5 M NaCl, heated at 65 °C for 4 h, then treated with protease K and further incubated at 45 °C for 1 h. The chromatin complex was purified by PCR purification kit. Then the purified immunoprecipitation DNA was used for qPCR, and the 2^−△△Ct^ method was used to calculate the p53 occupancy rate on the p21 promoter. The primer sequences of p53 binding site in p21 promoter were as follows: Fw 5′-GTTCCCAGCACTTCCTCTCC-3′; Re 5′-GAAGCAGGCAGCATAGGGAT-3′.

### Xenograft in nude mice

Four to Five-week-old Female BALB/c nude mice were purchased from the experimental Animal Center of the Tianjin Medical University (Tianjin, China) and maintained under pathogen-free conditions. All animal procedures were performed according to the requirements of animal welfare ethics and approved by the Laboratory Animal Management and Use Committee (IACUC) of Tianjin Medical University (TMUaMEC 2022041).

Xenograft in nude mice was performed as we reported before [19]. Mice were subcutaneously inoculated with CRC cells (1 × 10^7^ cells). After successful tumor bearing (about 5 mm in diameter), mice (n = 5) were randomly divided into the control group and HST treated group and intraperitoneally injected with saline or HST (20 mg/kg) once every other day for totally 18 days. The tumor volume was measured with calipers every other day, and calculated using the formula: (width^2^ × length)/2. The body weight was measured every other day. The nude mice were sacrificed at day 18 after the first injection. The tumor tissues were excised, and fixed with 4% paraformaldehyde for immunohistochemical detection.

### Hematoxylin–eosin (H&E) and immunohistochemical (IHC) staining

H&E and IHC staining were performed as reported [17, 19]. In brief, fixing, embedding, and slicing of the tumor tissues were conducted according to the classical procedures. For IHC staining, after deparaffinization using xylene, the slides were exposed with 3% hydrogen peroxide in methanol to quench the endogenous peroxidases, then blocked using bovine serum albumin (BSA) followed by an incubation with Ki-67 or p53 primary antibodies at 4 °C for 12 h, and then cultured with Bond Polymer (anti-rabbit poly-HRP-IgG) at room temperature for 1 h. Subsequently, the slides were treated with diaminobenzidine (DAB) peroxidase, counterstained with hematoxylin, and visualized under a microscope (BX51, Olympus, Japan).

### Statistical analysis

Experiments were conducted in triplicate. Data from three independent repeats were presented. The results were expressed as mean ± SD. Statistical analyses were determined by Student’s t test and one-way ANOVA using GraphPad software. *p* < 0.05 was regarded statistically significant.

## Results

### HST inhibits CRC cell growth independent of p53 status

**Fig. 1:**
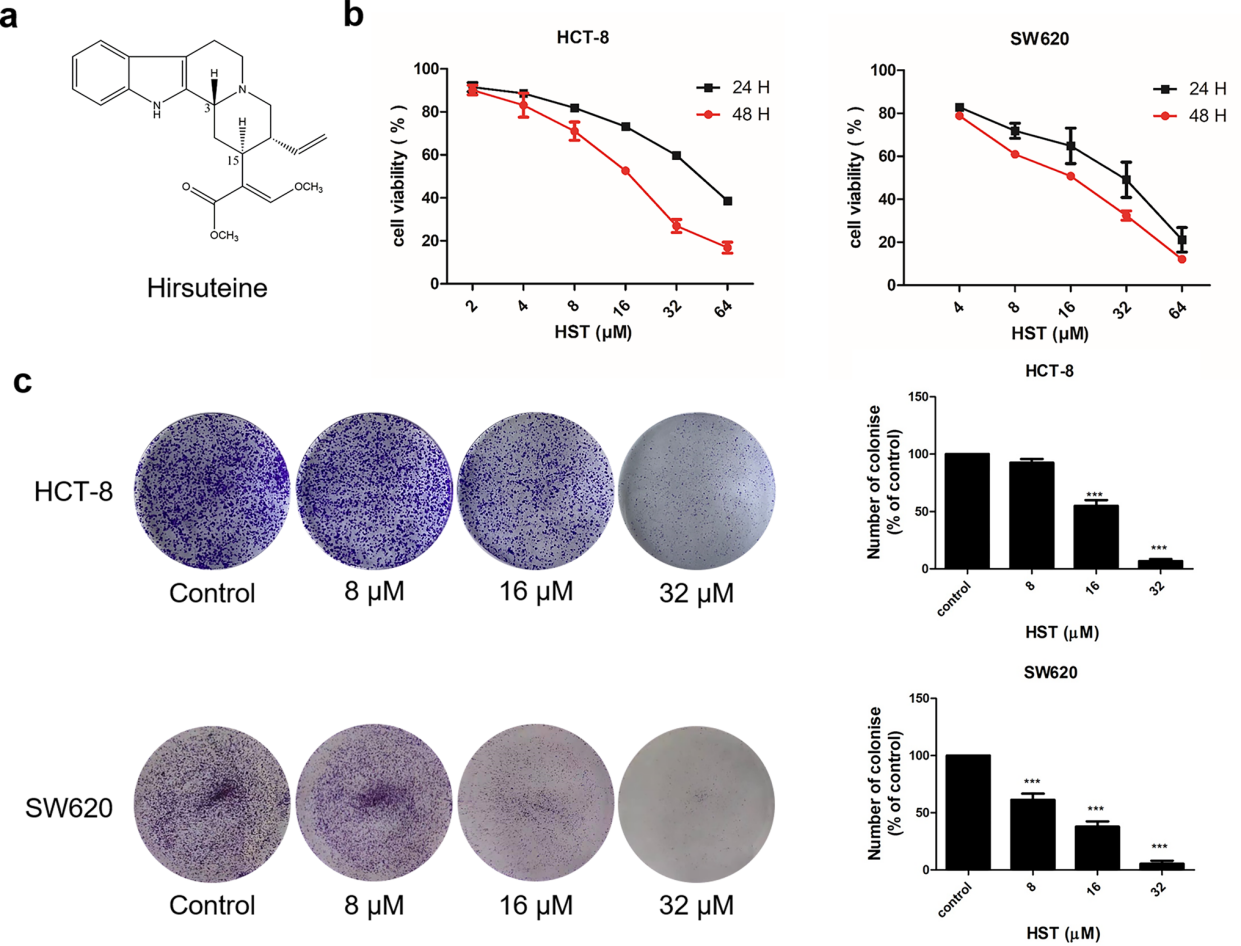
HST inhibits CRC cell proliferation independent of p53 status. **a** Structure of HST. **b** MTT assays. HCT-8 and SW620 cells were treated with indicated doses of HST for 24 h and 48 h, cell viability was evaluated by MTT assays. **c** Colony formation assays. HCT-8 and SW620 cells were treated with indicated doses of HST for 48 h, colony formation assays were conducted. Data are presented as mean value ± SD. ***p < 0.001 versus control

The chemical structure of HST was shown in Fig. 1a. MTT and colony formation assays were conducted to confirm the antiproliferative effect of HST on human CRC cells harboring different p53 status, HCT-8 (wtp53) and SW620 (mutp53^R273H^). As 5-fluorouracil (5-Fu) is the first-line chemotherapeutic drug for CRC, the antiproliferative effect of 5-Fu on HCT-8 and SW620 cells were firstly examined by MTT. The results showed that 5-Fu decreased cell viability of HCT-8 and SW620 cells, with IC_50_ values of 25.15 μM and 75.97 μM for 48 h treatment, respectively (Supplementary Fig. S1). Although 5-Fu moderately inhibited cell viability in both cell lines, SW620 cells seemed less sensitive to 5-Fu, with an IC_50_ more than threefold greater than that in HCT-8 after 48 h treatment. HCT-8 and SW620 cells were then treated with increasing doses of HST for 24 h and 48 h. Cell viability, evaluated by MTT assay, showed a sharp reduction in response to HST treatment in a dose-dependent manner, and HST inhibited cell viability of SW620 more potently than that of HCT-8. The IC_50_ values of HST in HCT-8 were 43.87 μM (24 h) and 16.05 μM (48 h), while the IC_50_ values of HST in SW620 were less, 24.68 μM (24 h) and 14.16 μM (48 h) (Fig. 1b). The results of colony formation assay also showed that HST efficiently inhibited the growth of HCT-8 and SW620 cells, as was evidenced by a decreased colony formation in both cell lines in response to increasing doses of HST (Fig. 1c). Notably, HST exerted greater proliferative inhibition on SW620 cells than on HCT-8 cells. These results revealed that HST decreased the proliferation of CRC cells independent of p53 status.

### HST promotes cell cycle arrest and cell autophagy on CRC cells

**Fig. 2:**
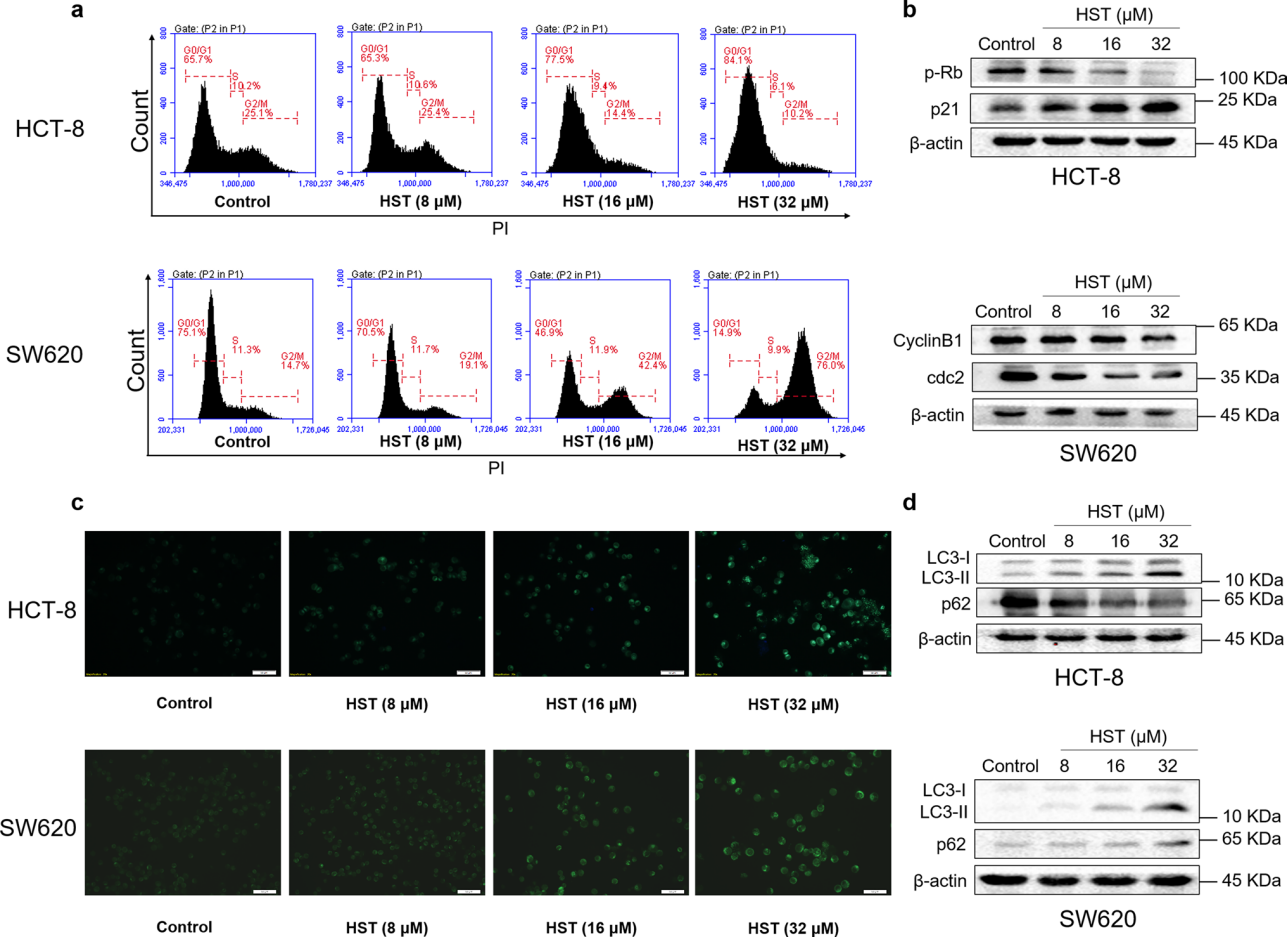
HST induces cell cycle arrest and cell autophagy on CRC cells. HCT-8 and SW620 cells were treated with indicated doses of HST for 48 h. **a** Cell cycle distribution was examined by Flow cytometry after PI staining. **b** Western blot analysis of cell cycle mediators, including p-Rb, p21, cdc2, cycline B1, was performed. **c** Cell autophagy was determined by MDC staining, cells were photographed. Scale bar: 50 μm. **d** Western blot analysis of cell autophagy related factors, LC3B and p62, was performed. All experiments were conducted at least three times, and representative data were shown

To investigate the mechanism by which HST inhibited cell growth, cell cycle distribution by flow cytometry and cell autophagy by MDC staining were conducted. The result in Fig. 2a showed that HST increased G0/G1 phase cell population in HCT-8 cells, whereas in SW620 cells, HST increased G2/M phase cell population, suggesting that HST treatment induced G0/G1 arrest in HCT-8 and G2/M arrest in SW620. Consistently, the results of the Western blot analysis in Fig. 2b showed that HST enhanced the expression of p21, while reducing p-Rb level in HCT-8 cells. In SW620 cells, HST treatment led to the reduction of cdc2 and cyclin B1. These results further reinforced the G0/G1 arrest and G2/M arrest effect of HST in HCT-8 and SW620 cells, respectively. To investigate cell autophagy, HCT-8 and SW620 cells were exposed to increasing doses of HST and stained with MDC. The results in Fig. 2c showed that HST induced significant formation of MDC puncta in both cell lines, suggesting induction of cell autophagy by HST. The results of Western blot analysis showed that, in HCT-8 cells, HST upregulated LC3-II, downregulated p62, while in SW620 cells, HST increased the level of LC3-II and p62 (Fig. 2d). To determine the role of autophagy induction by HST in cell death, MTT assay was conducted to evaluate the effect of 3-methyladenine (3-MA), an autophagy inhibitor, on proliferative inhibition effect of HST. The results in Supplementary Fig. S2 showed that 3-MA alleviated cell growth inhibition of HST on both cells, suggesting that HST induced cytostatic autophagy in HCT-8 and SW620 cells. Cell apoptosis was also examined and the results showed that, only in SW620 cells, a significant cell apoptosis promotion by HST was observed (Supplementary Fig. S3). These results further confirmed that HST exerted antiproliferative activity on CRC cells by promoting cell cycle arrest and inducing cell autophagy.

### Gene expression profile changes in HCT-8 cells by HST reveal a p53 signaling participation

**Fig. 3:**
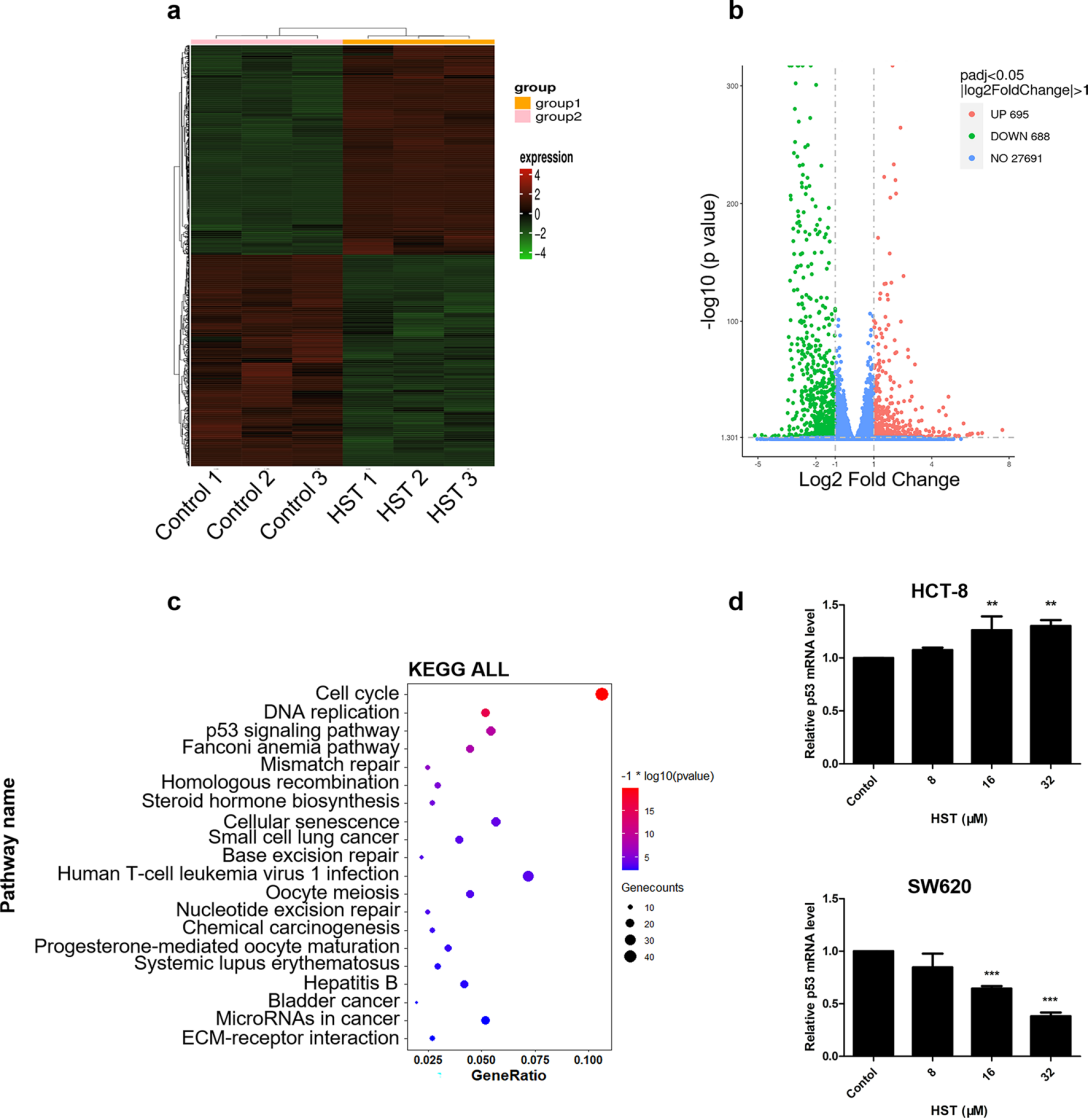
Gene expression profile changes in HCT-8 cells by HST using RNA-Seq. **a** Hierarchical clustering heat map of differentially expressed genes between HST (32 μM) treated HCT-8 cells and DMSO treated cells (Control). **b** The volcano plot of differential expressed genes in HST (32 μM) treated HCT-8 cells compared to the control. Red points represent significantly upregulated genes, whereas green points represent downregulated genes. Blue points are genes whose expression changed with relatively low or no statistical significance. Cut off is ∣log2 fold change∣ > 1, *p* < 0.05. **c** KEGG analysis. **d** mRNA expression level of p53 determined by qRT-PCR in HCT-8 and SW620 cells in response to HST for 48 h. Data are presented as mean value ± SD. **p < 0.01, ***p < 0.001 versus control

To explore the potential mechanism of anticancer efficacy of HST on CRC cells, RNA sequencing of HST-treated HCT-8 cells was conducted. DMSO treatment was used as control. HST exposure led to broad changes in gene expression, with 695 upregulated and 688 downregulated differentially expressed genes (Fig. 3a, b). KEGG analysis using the clusterProfiler R package (3.8.1) evidenced a strong activation of the p53 pathway (Fig. 3c). This result was further validated by qRT-PCR, in which HST treatment induced a significant upregulation of p53 mRNA expression in HCT-8 cells (Fig. 3d). While in SW620, a downregulation of p53 mRNA expression by HST was observed (Fig. 3d). Our findings suggested that the p53 pathway might participate in anticancer effect of HST on CRC cells.

### HST enhances wtp53 stabilization and transcriptional activity in HCT-8 cells

**Fig. 4:**
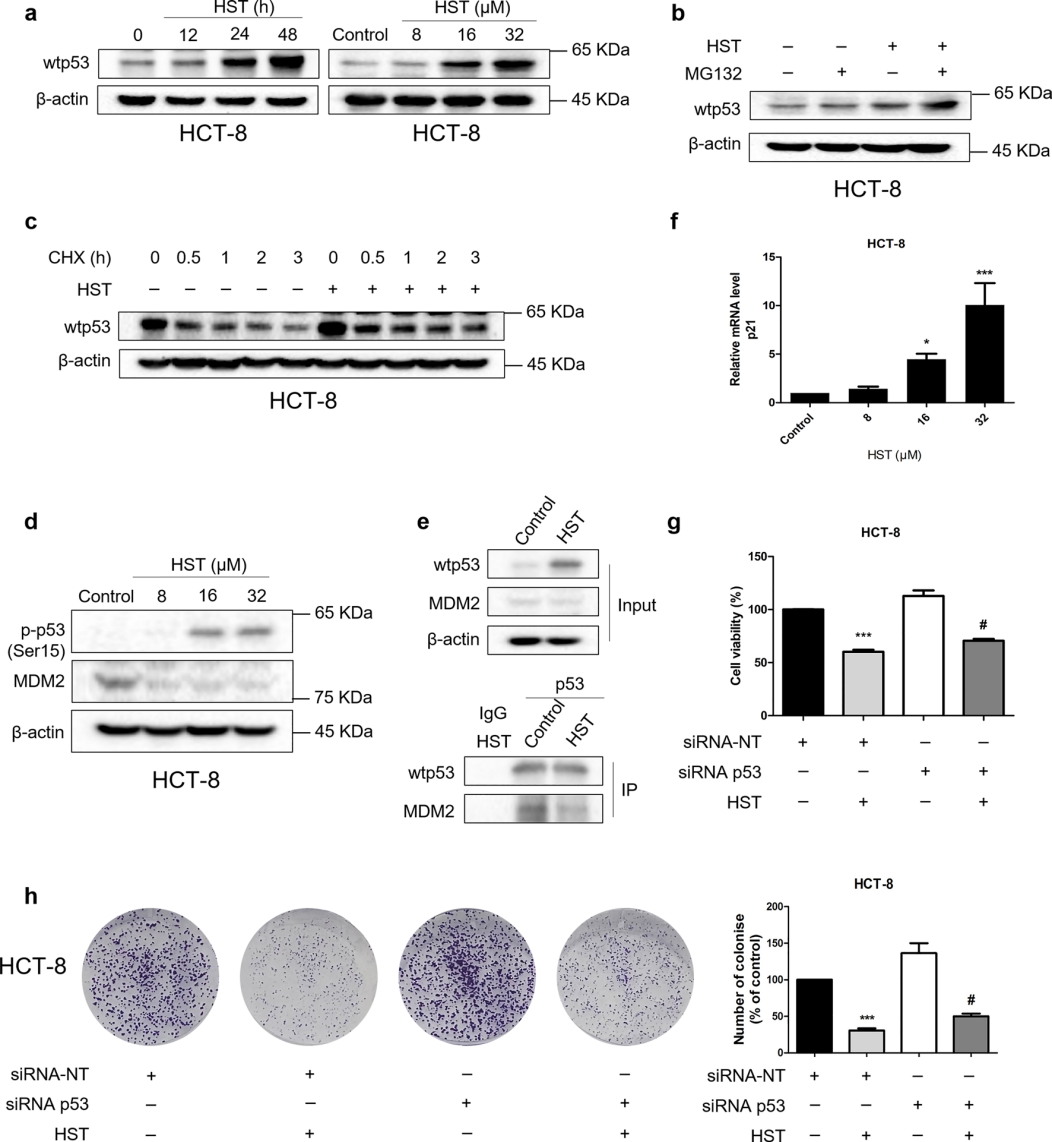
HST enhances p53 stabilization, transcriptional activity in HCT-8 cells. **a** HCT-8 cells were exposed with HST (8, 16, 32 μM) for 48 h or 32 μM HST for 0, 12, 24, 48 h, Western blotting was performed to detect wtp53 expression. **b** HCT-8 cells were treated with HST (32 μM), MG132 (10 μM), or their combination, Western blotting was performed. **c** Half-life of wtp53 protein was determined by CHX-chase assays. HCT-8 cells pretreated with 10 μM cycloheximide (CHX) for 0, 0.5, 1, 2, 3 h were exposed with or without HST (32 μM), Western blotting was performed. **d** HCT-8 cells were treated with HST (8, 16, 32 μM) for 48 h, p-p53 (Ser 15) and MDM2 levels were examined by Western blotting. **e** Co-IP. HCT-8 cells were treated with vehicle or HST (32 μM) for 48 h, cell lysates were immunoprecipitated with p53 antibody. Immunoprecipitates and inputs were immunoblotted with the indicated antibodies. **f** qRT-PCR. HCT-8 cells were treated with 8, 16, 32 μM HST for 48 h, mRNA level of p21 was examined by using qRT-PCR analysis. **g** Cell viability of HCT-8 cells treated with siRNA p53, HST or their combination was measured using MTT assay. siRNA non-targeting (siRNA-NT) was used as negative control. **h** Colony formation of HCT-8 cells treated with siRNA p53, HST (16 μM) or their combination was measured using colony formation assay. siRNA-NT was the negative control. Experiments were conducted at least three times, and representative data were shown. Data are presented as mean value ± SD. *p < 0.05, ***p < 0.001 versus control or siRNA-NT, ^#^*p* < 0.05 versus the combination treatment of siRNA-NT and HST

Wtp53 protein is a key cancer suppressor and usually halts cancer progression by mediating various cellular activities. However, wtp53 is commonly kept at a low level because it is constitutively ubiquitinated and proteasomally degraded by E3 ubiquitin ligase, such as MDM2 [8]. Since the result of RNA-seq of HST-treated HCT-8 cells revealed a p53 signaling participation, p53 protein expression of HCT-8 in response to HST at different doses and different time points was examined using Western blotting. The results in Fig. 4a showed that HST treatment induced a p53 accumulation in HCT-8 cells dose-dependently and time-dependently. When co-treated with MG132, a proteasome inhibitor, p53 accumulated to a higher level (Fig. 4b). Furthermore, we examined the effect of HST on p53 protein stability. HCT-8 cells were exposed with or without a protein synthesis inhibitor, cycloheximide (CHX), and p53 protein expression was evaluated by Western blotting. The result showed that the p53 protein half-life in HCT-8 cells was significantly prolonged by HST (Fig. 4c). The above results indicated that HST enhanced p53 stability and inhibited proteasomal degradation of p53 in HCT-8 cells. These findings were further confirmed by increasing phosphorylation at Ser 15 of p53 and decreasing MDM2 after HST exposure (Fig. 4d). Moreover, MDM2 was precipitated by p53, and a reduced interaction between MDM2 and p53 in HCT-8 cells after HST treatment was observed (Fig. 4e), suggesting that HST promoted p53 stability probably by inhibiting p53 degradation induced by MDM2. Transcriptional activity of p53 in HCT-8 after HST treatment was examined by qRT-PCR. The result showed that HST increased mRNA level of p21, a p53 downstream target, in HCT-8 cells (Fig. 4f). Moreover, siRNA p53 significantly impaired the growth inhibition of HST on HCT-8 cells (Fig. 4g, h, Supplementary Fig. S4). Taken together, these results demonstrated that HST enhanced wtp53 stabilization and transcriptional activity in HCT-8 cells, probably leading to the anticancer effect of HST in HCT-8 cells.

### HST decreases mutp53^R273H^ expression and restores wild-type-like properties to mutp53 ^R273H^ in SW620 cells

**Fig. 5:**
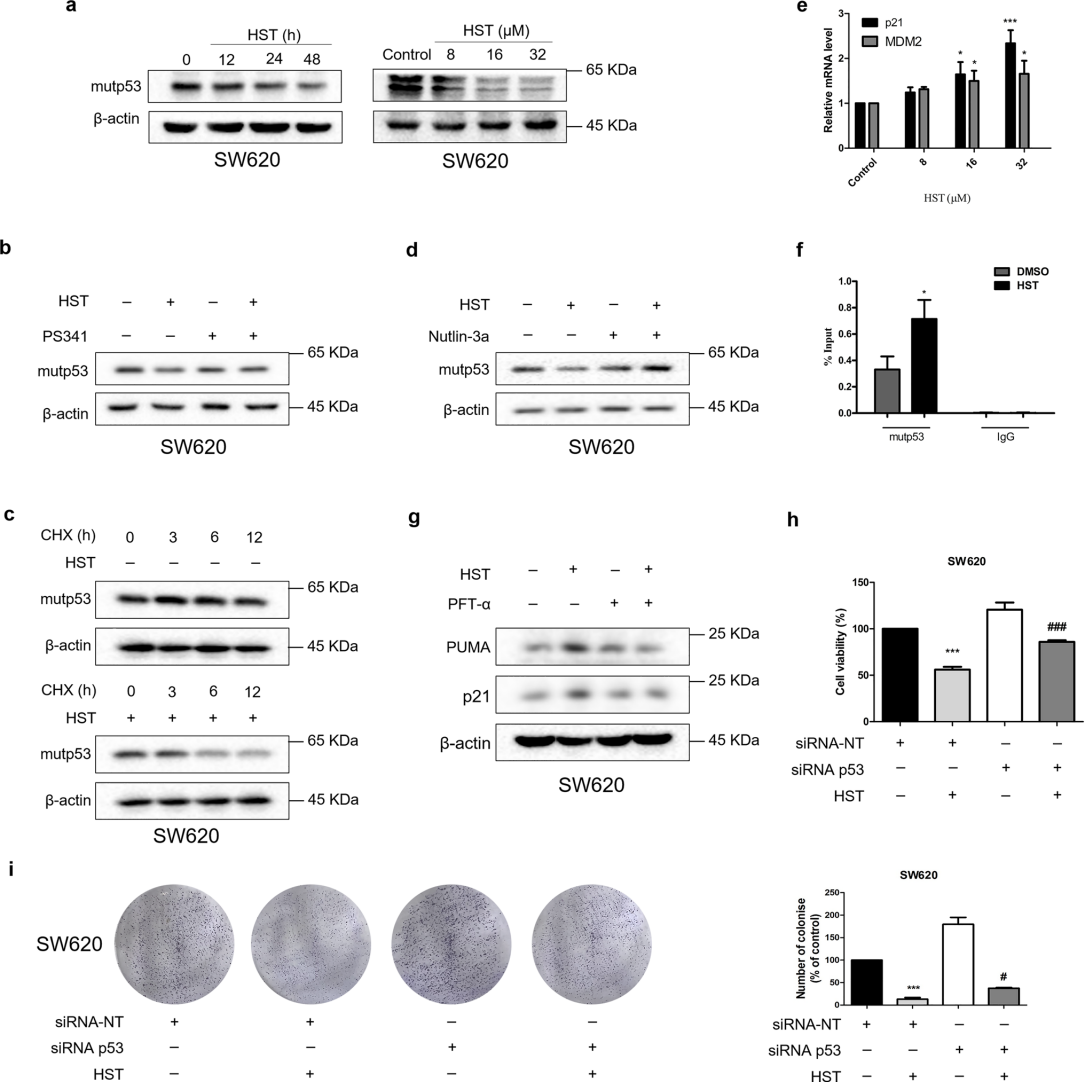
HST induces mutp53^R273H^ proteasomal degradation and restores its wild-type-like properties in SW620 cells. **a** SW620 cells were treated with HST (8, 16, 32 μM) for 48 h or 32 μM HST for 0, 12, 24, 48 h, Western blotting was performed to detect mutp53^R273H^ expression. **b** SW620 cells pretreated with 2 μM PS341 were exposed with DMSO or HST (32 μM), Western blotting was performed. **c** CHX-chase assays were conducted to detect the effect of HST on half-life of mutp53^R273H^ protein in SW620 cells. SW620 cells were treated with CHX (50 μM) alone or CHX combined with HST (32 μM) for 0, 3, 6, 12 h, western blotting was performed. **d** SW620 cells were treated with HST (32 μM), Nutlin-3a (15 μM) alone or in combination, mutp53^R273H^ protein level was examined by western blotting. **e** qRT-PCR. SW620 cells were treated with indicated doses of HST for 48 h, mRNA expressions of p53 transcriptional targets, p21 and MDM2, were detected by qRT-PCR. **f** ChIP assay. SW620 cells were treated with DMSO or HST (32 μM) for 48 h, cell lysates were immunoprecipitated with p53 antibody or IgG antibody (Negative control), DNA-binding ability of mutp53^R273H^ at the p21 promoter was determined by ChIP. **g** SW620 cells were treated with HST (32 μM), PFT-α (10 μM) alone or in combination, p21 and PUMA protein levels were examined by Western blotting. **h** Cell viability of SW620 cells treated with siRNA p53, HST or their combination was analyzed using MTT assay. siRNA negative control (siRNA-NT) was used as control. **i** Colony formation of SW620 cells treated with siRNA p53, HST (16 μM) or their combination was measured using colony formation assay. siRNA-NT was the negative control. Experiments were conducted at least three times, and representative data were shown. Data are presented as mean value ± SD. *p < 0.05, ***p < 0.001 versus control or siRNA-NT, ^#^*p* < 0.05, ^###^*p* < 0.001 versus the combination treatment of siRNA-NT and HST

*TP53* is one of the most frequently mutated genes in human cancers. In cancer cells, mutp53 protein often accumulates to a high level and harbors GOF activities instead of cancer-suppressive effects, ultimately promoting cancer progression [6]. To explore whether the mechanisms of HST-induced growth inhibition on HCT-8 and SW620 cells are similar, the effect of HST on mutp53^R273H^ protein expression was evaluated. SW620 cells were treated with increasing doses of HST for 48 h or 32 μM HST for 0, 12, 24, 48 h, and then Western blotting was performed. The result showed that, in SW620 cells, HST decreased mutp53^R273H^ expression in a dose- and time-dependent manner (Fig. 5a). Notably, this reduction was significantly reversed by proteasome inhibitors, PS341 and MG132 (Fig. 5b, Supplementary Fig. S5). The half-life of mutp53^R273H^ in SW620 cells was further examined by CHX-chase analysis. Upon the inhibition of protein synthesis by CHX, HST treatment shortened mutp53^R273H^ half-life, suggesting that the accumulation of mutp53^R273H^ in SW620 was inhibited by HST (Fig. 5c). To explore whether the decreased stability of mutp53^R273H^ by HST resulted from MDM2-mediated proteasomal degradation, an MDM2 inhibitor, nutlin-3a was introduced. The result in Fig. 5d showed that nutlin-3a totally impaired the effect of HST on mutp53^R273H^ stability, demonstrating that HST induced the MDM2-mediated proteasomal degradation of mutp53^R273H^ in SW620 cells. As transcriptional restoration is one of the strategies to solve mutp53 accumulation in cancer therapy [8], restoration of wild-type-like properties to mutp53 ^R273H^ in SW620 cells was examined by qRT-PCR and ChIP assays after SW620 cells were treated with different doses of HST for 48 h. The result in Fig. 5e showed that mRNA levels of p53 transcriptional targets, such as p21 and MDM2, were upregulated by HST exposure. Through ChIP assay, it was observed that HST increased the DNA-binding ability of mutp53^R273H^ at the p21 promoter in SW620 cells (Fig. 5f). Moreover, PFT-α, a p53 transcriptional inhibitor, reversed the transcriptional enhancement of HST on p53 downstream targets, p21 and PUMA, in SW620 cells, further confirming that HST might cause reactivation of mutp53^R273H^ (Fig. 5g). Furthermore, mutp53^R273H^ knockdown by siRNA significantly abrogated the inhibitory effect of HST on SW620 cells (Fig. 5h, i, Supplementary Fig. S4). Collectively, these results demonstrated that HST induced mutp53^R273H^ degradation and restored its wild-type-like properties in SW620 cells, which are closely related to the anticancer activity of HST.

### HST exerts inhibition effect against CRC through opposite regulation of wtp53 and mutp53^R273H^ in vivo

**Fig. 6:**
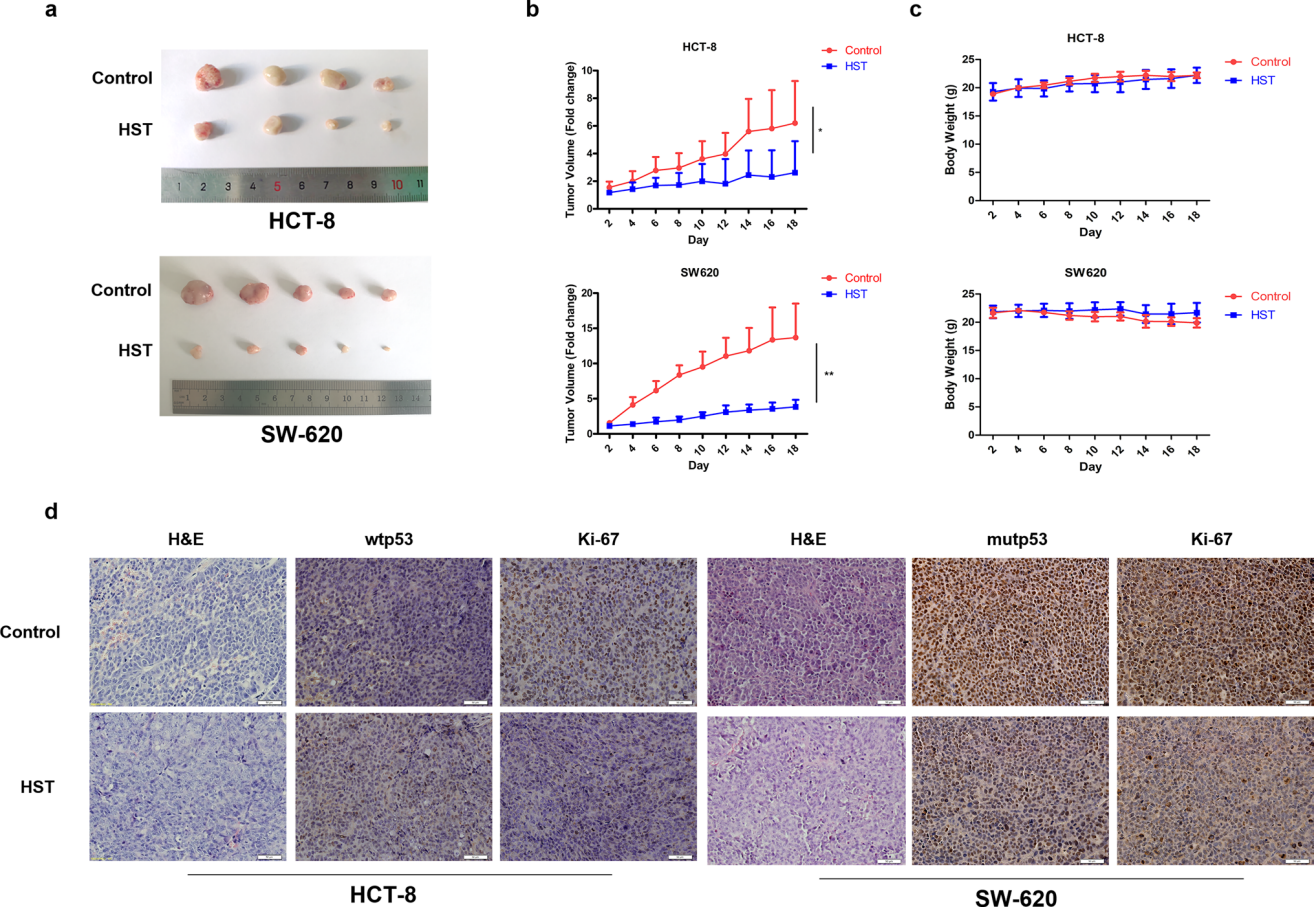
Anticancer effect of HST against CRC in vivo. **a** Photograph of excised HCT-8 and SW620 tumor xenografts. Nude mice implanted with HCT-8 or SW620 cells were intraperitoneal injected with saline (vehicle) or HST (20 mg/kg/day) every other day for totally 18 days. Top: vehicle-treated tumors. Bottom: HST-treated tumors. **b** Tumor volume of mice treated with HST and vehicle was monitored. **c** Body weight of mice treated with HST and vehicle was monitored. **d** H&E staining, Ki-67 and p53 expressions in HCT-8 and SW620 tumor xenografts treated with HST or vehicle by IHC analysis. Scale bar: 50 μm. Experiments were conducted at least three times, and representative data were shown. Data are presented as mean value ± SD. *p < 0.05, **p < 0.01 versus control

To investigate the anticancer effect as well as the underlying mechanism of HST against CRC in vivo, subcutaneous xenografts in nude mice using HCT-8 or SW620 cells were established. Compared with the vehicle-treated mice, HST-treated mice at a dose of 20 mg/kg/day for 18 days showed significantly suppressed tumor growth, resulting in 2.5-fold and 3.5-fold reduction in the tumor volume of HCT-8 and SW620 xenografts, respectively (Fig. 6a, b). Notably, this reduction by HST was not followed by body weight loss (Fig. 6c), suggesting a low systemic toxicity. The expression of p53 and Ki-67 in Xenografts were analyzed by IHC. The results in Fig. 6d showed that treatment with HST induced a significant downregulation of Ki-67 in both HCT-8 and SW620 xenografts, which further demonstrated the growth inhibitory efficacy of HST in vivo. Furthermore, an upregulation of wtp53 expression in HCT-8 xenografts and a downregulation of mutp53^R273H^ expression in SW620 xenografts caused by HST were observed. These results suggested that HST inhibits CRC cells growth in vivo probably through opposite regulation of wtp53 and mutp53^R273H^.

## Discussion

Clinical studies have shown that CRC patients with mutp53 display unsatisfactory response to 5-Fu which is commonly used in CRC therapy, and p53 mutation status is closely associated with CRC outcomes [7]. Consistently, our present study demonstrated that SW620 cells harboring mutp53^R273H^ showed less sensitivity to 5-Fu than HCT-8 cells expressing wtp53. However, both cell types were sensitive to HST. Compared with 5-Fu, HST more potently inhibited HCT-8 and SW620 cell proliferation with lower IC_50_ values. More importantly, HST exerted greater proliferative inhibition on SW620 cells than on HCT-8 cells, evidenced by the lower IC_50_ value of HST on SW620, suggesting that HST might be a promising CRC drug candidate.p53 is usually expressed at a relatively low level or is functionally inactivated in patients with wtp53 through transcriptional or posttranscriptional regulation mechanisms, thereby resulting in loss of cancer suppression function [20]. However, mutp53, frequently observed in various cancers, often shows accumulation and exerts GOF activities in cancer cells, contributing to cancer progression [6]. Importantly, studies have demonstrated that the prolonged stability of mutp53 protein is a critical factor for its GOF activities [5]. Thus, targeting the p53 pathway, including by stabilizing wtp53 or degrading mutp53, is believed to be a promising approach for the development of therapeutics against various cancers, including CRC. In the present study, we found that HST activated wtp53 in HCT-8 cells and depleted mutp53^R273H^ in SW620 cells, showing potent anticancer efficacy in CRC. Moreover, our results showed that HST arrested cell cycle of HCT-8 and SW620 at different phases. Wtp53 is essential to the G0/G1 checkpoint activation and cell autophagy promotion upon stress [21]. Consistently, in HCT-8 cells expressing wtp53, HST arrested cell cycle at G0/G1 phase and induced cell autophagy, which was associated with stability enhancement and transcriptional activation of wtp53 by HST. However, in SW620 cells, HST induced cell cycle arrest at G2/M phase. The different effect of HST on cell cycle distribution of HCT-8 and SW620 could be ascribed to cell context, as cancer cells harboring mutp53 are G1 checkpoint-deficient [21].

Factually, researchers have pointed out that wtp53 activation in cancer might exhibit undesirable toxicity because of p53 activation in normal cells [22]. However, others have also demonstrated that normal tissues are not significantly affected by p53 genetic re-establishment [23]. Interestingly, the low toxicity in mice and dual targeting at wtp53 and mutp53^R273H^ distinguish HST from other p53-targeted inhibitors, such as MDM2 inhibitor, which promotes restoration of wtp53 only. Our findings suggested that HST is worthy of further development with its own unique advantage in treating CRC with a different p53 status.

Mutp53, characterized by an increased half-life, often stabilizes and accumulates to high levels in cancer cells. Studies have indicated that the hyperstability of mutp53 might be due to a lack of ubiquitination [24]. It has been widely known that MDM2, a p53-specific E3 ubiquitin ligase, can induce proteasomal degradation of both wtp53 and mutp53 [25]. Besides MDM2, according to various reports, some other ubiquitin ligases, such as carboxyl terminus of Hsc70-interacting protein (CHIP) and constitutive photomorphogenesis protein 1 (COP1), also participate in p53 proteasomal degradation [24], but mutp53 can inhibit the activity of these ubiquitin ligases via forming stable complexes with them and escape ubiquitination and degradation. Our results suggested that, in SW620 cells, HST decreased the half-life of mutp53^R273H^, and led to mutp53^R273H^ downregulation. This reduction was reversed by proteasome inhibitors (PS341, MG132) and MDM2 inhibitor (nutlin-3a), respectively, suggesting that HST induced the MDM2-mediated proteasomal degradation of mutp53^R273H^ in SW620 cells. Furthermore, mutp53^R273H^ knockdown by siRNA significantly impaired the proliferation inhibition of HST on SW620 cells. Thus, it can be anticipated that depleting mutp53^R273H^ and restoring wild-type-like activities to mutp53^R273H^ by HST probably in part accounts for the anticancer effect of HST on SW620 cells.

Mutp53 ^R273H^ has been reported to play suppressive role in cell autophagy, which is thought to be one of the reasons for mutp53 oncogenic functions [26]. Furthermore, it has been reported that autophagy might be a part of mutp53 depletion, and some molecules such as capsaicin, PRIMA-1, and suberoylanilide hydroxamic acid can induce mutp53 degradation via an autophagic mechanism [27, 28]. In the current study, we found that HST promoted cell cytostatic autophagy in SW620 cells, evidenced by MDC staining and Western blotting. However, p62, which is a substrate of autophagy, was increased in SW620 cells after HST treatment, revealing that autophagy promotion by HST in SW620 was impaired. It has been documented that p62 plays important roles in protein degradation either through autophagy and lysosomal targeting or through ubiquitinate proteasomal degradation [29]. Notably, a recent study has described that the mutp53^R273H^-p62 axis participates in driving the ubiquitin-dependent proteasomal degradation of cell junction proteins, such as connexin 43, resulting in cancer cell migration and invasion in PANC-1 cells [30]. Thus, questions of whether autophagy promotion by HST is related to mutp53^R273H^ degradation, as well as the mechanism of impaired autophagy induced by HST on SW620 cells, still need to be elucidated in the future.

## Conclusion

In summary, our findings suggest that HST possesses anticancer efficacy against CRC via opposite modulation of wtp53 and mutp53^R273H^ in vitro and in vivo, illustrating the potential of HST to be a promising CRC drug candidate.

## Supplementary Information

Additional file 1

## Data Availability

The datasets for the current study are available from the corresponding author on reasonable request.
