# Supplementary material for: Anticancer efficacy of hirsuteine against colorectal cancer by opposite modulation of wild-type and mutant p53
Source: Discov Oncol. 2023 May 31;14:84. doi: 10.1007/s12672-023-00688-1 (PMC10232392; doi:10.1007/s12672-023-00688-1)
Supplement: Supplementary file 1 — Additional file 1 [file 12672_2023_688_MOESM1_ESM.docx]

**Anticancer efficacy of hirsuteine against colorectal cancer by opposite modulation of wild-type and mutant p53**

Yan Zhang^1, †^, Tingting Guo^1, †^, Shurong Li^1^, Zehao Ren^1^, Shan Gao^1^, Hao Lu^1^, Xuelan Ma^1^, Donghui Liu^1^, Yao Liu^2^, Dexin Kong^1, 🖂^, Yuling Qiu^1, 🖂^

^1^Tianjin Key Laboratory on Technologies Enabling Development of Clinical Therapeutics and Diagnostics, School of Pharmacy, Tianjin Medical University, Tianjin, China

^2^Department of Otorhinolaryngology Head and Neck Surgery, Tianjin First Central Hospital, Tianjin, China

🖂 Yuling Qiu, [qiuyuling@tmu.edu.cn](mailto:qiuyuling@tmu.edu.cn); Dexin Kong, [kongdexin@tmu.edu.cn](mailto:kongdexin@tmu.edu.cn)

**^†^** These authors have contributed equally to this work.

**Supplementary Figures**

**Supplementary Fig. 1**


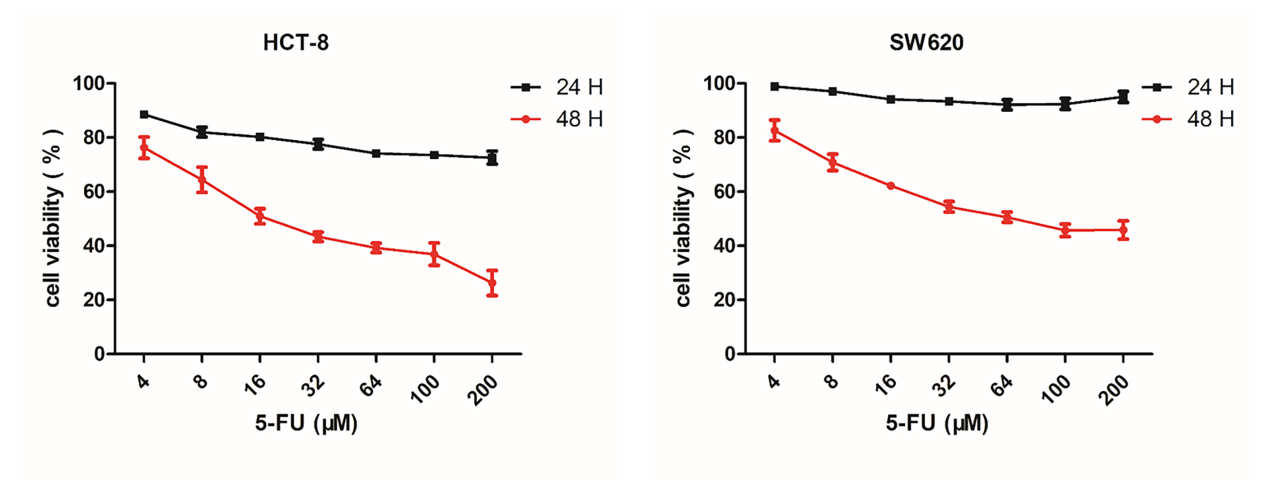


**Supplementary Fig. 1** MTT assays. HCT-8 and SW620 cells were treated with indicated doses of 5-Fu for 24 h and 48 h, cell viability was evaluated by MTT assays.

**Supplementary Fig. 2**


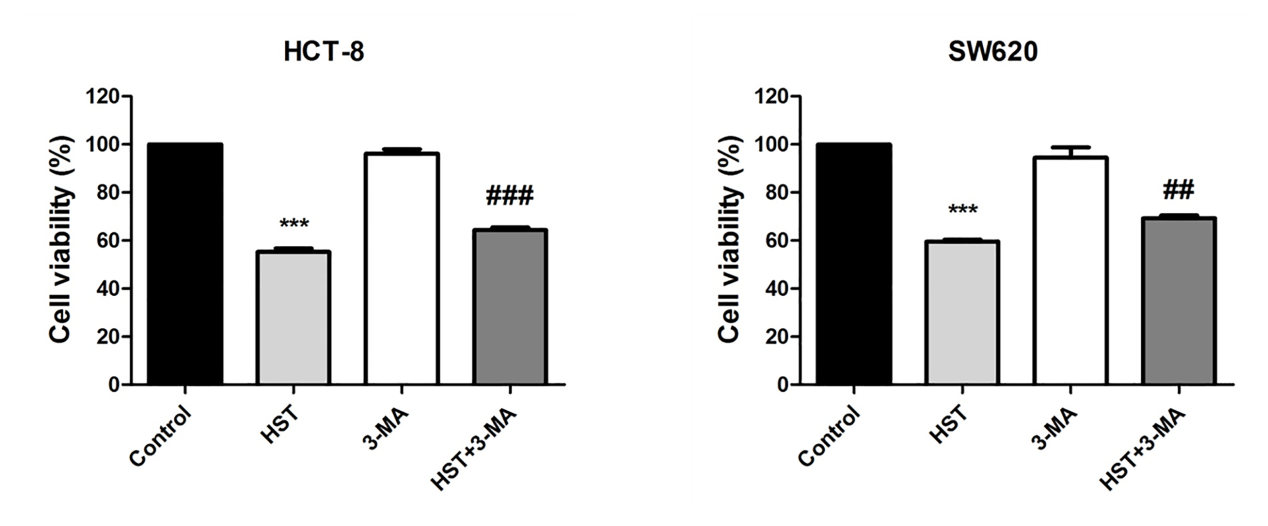


**Supplementary Fig. 2** Role of HST-induced autophagy in cell death. HCT-8 and SW620 cells were treated with HST (32 µM) and/or 3-MA (100 μM) for 48 h. Cell viability was determined by MTT. Data are presented as mean value ± SD. ^***^p < 0.001 versus control, ^##^p < 0.01, ^###^p < 0.001 versus HST treatment.

**Supplementary Fig. 3**


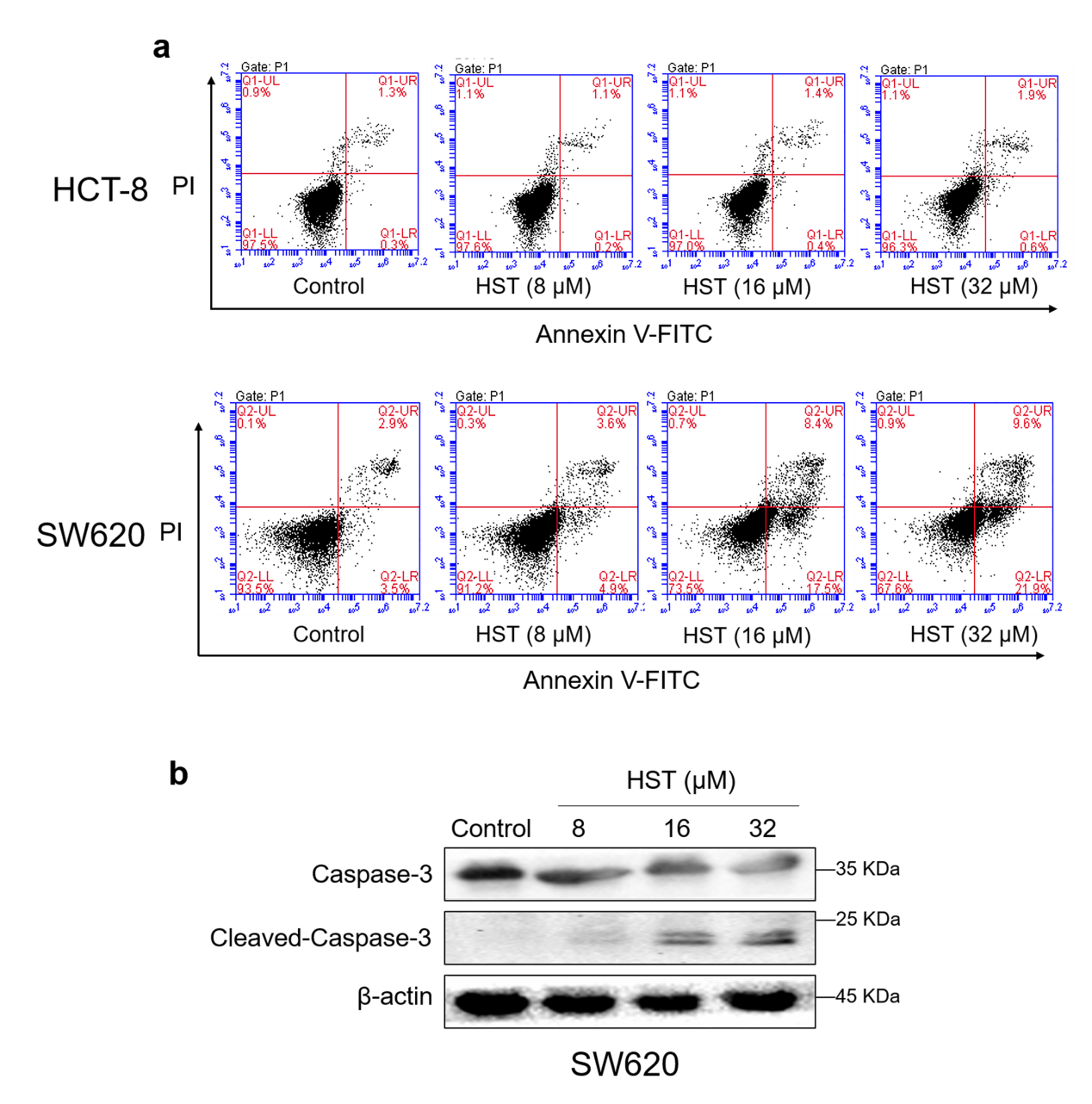


**Supplementary Fig. 3** Cell apoptosis. HCT-8 and SW620 cells were treated with indicated doses of HST for 48 h. **a** cell apoptosis was examined by Flow cytometry after Annexin V/PI staining. **b** Western blot analysis of Caspase-3 in HST treated SW620 cells was performed.

**Supplementary Fig. 4**


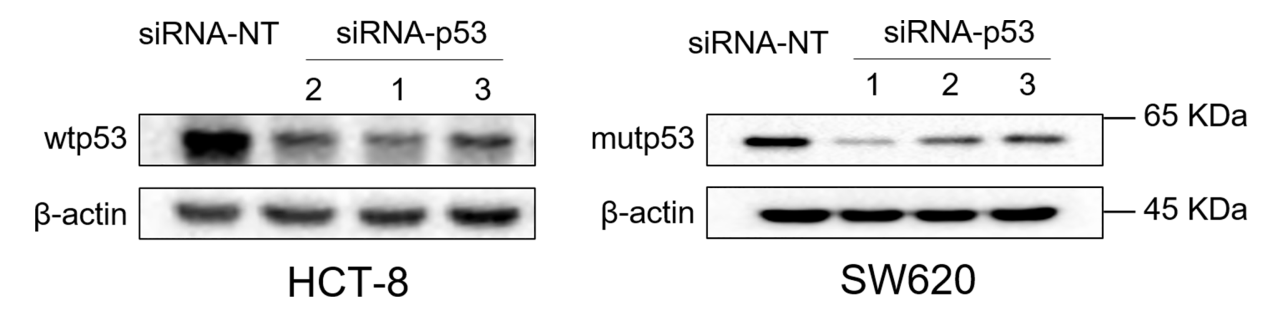


**Supplementary Fig. 4** Determination of p53 knockdown by siRNA. HCT-8 and SW620 cells were transfected with non-targeting siRNA or siRNA p53. Western blotting was conducted to determine p53 expression level.

**Supplementary Fig. 5**


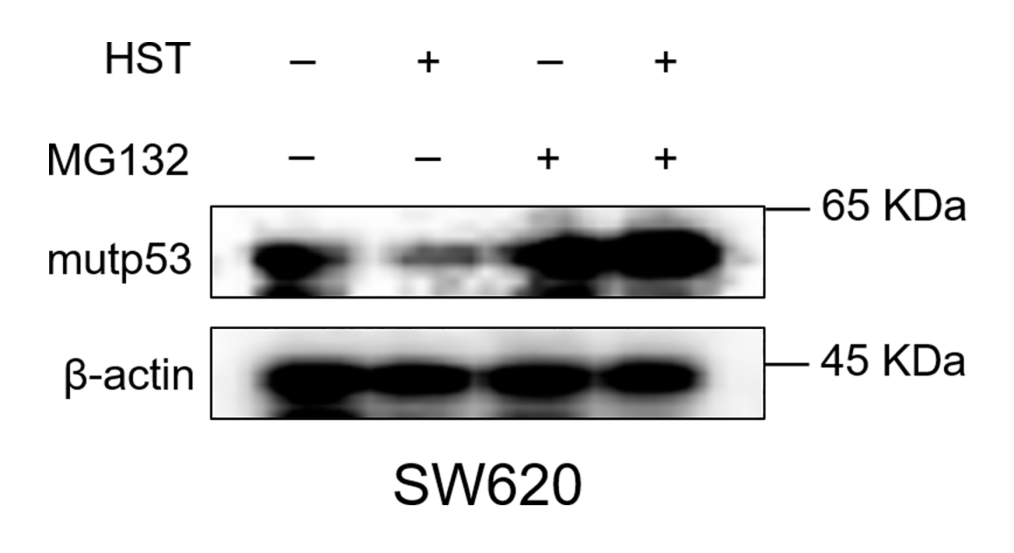


**Supplementary Fig. 5** HST induces mutp53^R273H^ proteasomal degradation. SW620 cells pretreated with MG132 (2 μM) were treated with DMSO or HST (32 μM), western blotting was conducted to evaluate mutp53^R273H^ expression level.
